# Genetic Diversity of Landraces and Improved Varieties of Rice (*Oryza sativa* L.) in Taiwan

**DOI:** 10.1186/s12284-020-00445-w

**Published:** 2020-12-14

**Authors:** Ai-ling Hour, Wei-hsun Hsieh, Su-huang Chang, Yong-pei Wu, Han-shiuan Chin, Yann-rong Lin

**Affiliations:** 1 Department of Life Science, Fu-Jen Catholic University, New Taipei City, 242062 Taiwan; 2 Department of Agronomy, National Taiwan University, Taipei, 10617 Taiwan; 3 Department of Agronomy, Chiayi Agricultural Experiment Branch, Taiwan Agricultural Research Institute, Chiayi, 600015 Taiwan

**Keywords:** Genetic diversity, Landraces, Rice, Taiwan

## Abstract

**Background:**

Rice, the most important crop in Asia, has been cultivated in Taiwan for more than 5000 years. The landraces preserved by indigenous peoples and brought by immigrants from China hundreds of years ago exhibit large variation in morphology, implying that they comprise rich genetic resources. Breeding goals according to the preferences of farmers, consumers and government policies also alter gene pools and genetic diversity of improved varieties. To unveil how genetic diversity is affected by natural, farmers’, and breeders’ selections is crucial for germplasm conservation and crop improvement.

**Results:**

A diversity panel of 148 rice accessions, including 47 cultivars and 59 landraces from Taiwan and 42 accessions from other countries, were genotyped by using 75 molecular markers that revealed an average of 12.7 alleles per locus with mean polymorphism information content of 0.72. These accessions could be grouped into five subpopulations corresponding to wild rice, *japonica* landraces, *indica* landraces, *indica* cultivars, and *japonica* cultivars. The genetic diversity within subpopulations was: wild rices > landraces > cultivars; and *indica* rice > *japonica* rice. Despite having less variation among cultivars, *japonica* landraces had greater genetic variation than *indica* landraces because the majority of Taiwanese *japonica* landraces preserved by indigenous peoples were classified as *tropical japonica*. Two major clusters of *indica* landraces were formed by phylogenetic analysis, in accordance with immigration from two origins. Genetic erosion had occurred in later *japonica* varieties due to a narrow selection of germplasm being incorporated into breeding programs for premium grain quality. Genetic differentiation between early and late cultivars was significant in *japonica* (*F*_ST_ = 0.3751) but not in *indica* (*F*_*ST*_ = 0.0045), indicating effects of different breeding goals on modern germplasm. Indigenous landraces with unique intermediate and admixed genetic backgrounds were untapped, representing valuable resources for rice breeding.

**Conclusions:**

The genetic diversity of improved rice varieties has been substantially shaped by breeding goals, leading to differentiation between *indica* and *japonica* cultivars. Taiwanese landraces with different origins possess various and unique genetic backgrounds. Taiwanese rice germplasm provides diverse genetic variation for association mapping to unveil useful genes and is a precious genetic reservoir for rice improvement.

## Background

Asian cultivated rice (*Oryza sativa* L.), feeding more than 90% of the human population in Asia, is one of the world’s most important crops. Wild ancestors and landraces with rich genetic diversity and wide adaptation to various environments provide valuable and useful genetic resources for crop improvement (Dwivedi et al. 2016; Kovach and McCouch 2008; Sang and Ge 2013). Natural germplasm preserved by in situ and/or ex situ conservation is in urgent need of systematic evaluation to unveil new genes or alleles to incorporate into breeding programs for crop improvement. For rice, the most well-known germplasm conservation center is the International Rice Genebank Collection at the International Rice Research Institute (IRRI). Single nucleotide polymorphisms (SNPs) and structural variants revealed by resequencing accelerate research on genetic diversity, evolution, association studies of genotypes and phenotypes and allele mining (Huang et al. 2010; Li et al. 2014; Wang et al. 2018; Zhao et al. 2018).

*Oryza sativa* was domesticated from wild rice, *O. nivara* or *O. rufipogon* (Chang 1976; Khush 1997), and two distinct varietal groups, ssp. *indica* and ssp. *japonica*, are well recognized and dated as Hsien (秈) and Keng (稉), respectively, in the Hang dynasty about 2000 years ago (Chou 1948; Wang et al. 2018). Distinct morphology and post-reproductive barriers between ssp. *indica* and ssp. *japonica* were first thought to have resulted from independent domestication by different ancient populations or a single domestication with multiple origins recently (Londo et al. 2006; Choi et al. 2017; Choi and Purugganan 2018). The genetic diversity of *O. sativa* was dramatically reduced by bottleneck effects of selective sweeps in early domestication (Caicedo et al. 2007; Kovach and McCouch 2008). Landraces which are morphologically recognizable and have historical origins exhibit lower genetic diversity than wild relatives but higher than modern cultivars because of adaptation to local environments and diversity of farmers’ preferences (Pusadee et al. 2009; Thomson et al. 2007). The allele richness of landraces was, in general, about 30% higher than that of cultivars (Kovach and McCouch 2008; Zhang et al. 2009) and landraces possess a wealth of abiotic tolerances, biotic resistances and other superior characters. Taken together, investigation of morphological, physiological, and genetic diversity of landraces will provide valuable information and resources for modern rice breeding.

In Taiwan, archaeological evidence shows that rice has been cultivated by indigenous people for more than 5000 years (Hu 1993). Excavated grains exhibit various sizes and shapes and resemble tropical *japonica* and *indica* rice (Hsieh et al. 2011)*.* In the early seventeenth century, immigrants from two provinces of southeast China, Fujian and Guangdong, brought *indica* landraces to Taiwan. In the late nineteenth century, approximately 1197 collections of *temperate* *japonica* rice were introduced from Japan (Iso 1964). Sixty Taiwanese landraces have been widely used in rice research and breeding, revealed by 16 domestication-syndrome genes (Hsieh et al. 2011). Taiwanese landraces have contributed significantly to modern *indica* and temperate *japonica* rice breeding in Asian countries. The most renowned example is IR8, the miracle rice with high yield that mitigated a food crisis in the 1970s and evoked a Green Revolution in Asia, which inherited the semi-dwarf allele (*sd1*) from Taiwanese landrace Dee-Geo-Woo-Gen (DGWG) (Evenson and Gollin 2003). Indeed, the DGWG allele has been extensively applied to improve grain yield of both *indica* and *japonica* varieties in the past 50 years (Sasaki et al. 2002; Asano et al. 2011; Zhao et al. 2010). Taichung 65 (TC65), an old *japonica* cultivar, inherited null alleles of two photoperiodic genes, *Ehd1* and *Hd1*, from landraces and has been extensively applied in modern rice breeding and in studying flowering in response to day length (Doi et al. 2004; Hsieh et al. 2011; Lin 1991; Wei et al. 2016; Yano et al. 2000).

Many modern cultivars integrate *temperate japonica* and *indica* rice toward meeting the major demands of daily dining and traditional food processing in Taiwan. A lot of genetic variation is found in Taiwanese rice germplasm because of natural selection for adaptation to various environments, noting that Taiwan encompasses tropical and subtropical zones in a broad range of altitudes (0–3952 m). The genetic diversity of Taiwanese rice germplasm, originating from different geographic areas and admixed by humans in different epochs, is expected to be high (Chin et al. 2016).

To unravel admixing of rice germplasm imposed by natural and artificial selection is important for basic scientific research and breeding, each relying on information about genetic diversity and population structure. In this study, a diversity panel of 148 accessions, including 53 modern varieties, 83 landraces, and 12 wild rice originating from Taiwan, Japan, China and countries of southeast Asia and south Asia, was genotyped with 75 markers to assess genetic diversity and population structure, conducting principal coordinate analysis (PCoA) and producing a phylogenetic tree. In addition, Taiwanese landraces are scrutinized, gaining insight into their significant roles in genetic and breeding research.

## Results

### Genetic Diversity of Polymorphic Markers

A total of 953 alleles were detected from 75 DNA markers, including 49 simple sequence repeat (SSR), 6 sequence-tagged site (STS) and 20 indel markers, across the diversity panel of 148 rice accessions, including 12 wild rices, 83 landraces, 24 *indica* cultivars, and 29 *japonica* cultivars (Additional file 1: Table S1). The allele number per locus ranged from 3 to 37 with an average of 12.7, and the majority of markers revealed 6–15 alleles. (Additional file 2: Figure S1a, Additional file 1: Table S2). Eight markers, RM472, RM2334, RM4108, CH0509, P17G10–24, RM1761, RM4154 and RM5708, were highly polymorphic with more than 20 alleles detected (Additional file 1: Table S2). Polymorphism information content (PIC) values ranged from 0.18 to 0.95 with an average of 0.72, and 66 (88%), 8 (10.7%) and 1 (1.3%) markers were highly, moderately and slightly informative, with PIC ≥0.5, 0.5 > PIC ≥0.25, and < 0.25, respectively (Additional file 2: Figure S1b, Additional file 1: Table S2). Overall, these 75 markers provided plentiful allele information to assess genetic diversity, population structure, and genetic distances of this rice diversity panel.

### Genetic Structure and Diversity of Subpopulations

These 148 accessions could be divided into two subpopulations according to inferred population structures, with*ΔK* values found to be highest at *K* = 2 by STRUCTURE analysis (Additional file 2: Figure S2). The japonica group constituted 62 accessions, including one wild rice, *O. rufipogon*, 29 Taiwanese *japonica* cultivars and 32 landraces from Taiwan, Japan, and China. All 29 Taiwanese *japonica* cultivars, except Kaohsiung 145, shared 99.9% ancestry, indicating a consistent genetic background. The indica group contained 86 accessions, including 24 cultivars, 51 landraces, and 11 wild rices. Admixture, simulated single genetic background less than 80%, was frequently observed in wild rice, except *nivara*-2 and *rufipogon*-21, but occasionally in cultivars (Taichung Sen 17 and Basmati 385) and landraces (Tangengenrankatsu and Chin-Men-Tou-Men-Hung-Mi) (Additional file 2: Figure S3a).

To further subdivide this germplasm, five subpopulations, denoted PopI to PopV, were obtained as *K* = 5 was the optimum number of subpopulations identified by STRUCTURE analysis (Table 1, Additional file 2: Figure S3b). The japonica group was subdivided into two subpopulations, PopII and PopIII. Most *japonica* landraces were grouped into PopII, including 23 and 2 accessions originating from Taiwan and China, respectively. PopIII contained 36 *japonica* accessions, including 29 cultivars sharing 89–99% uniform subpopulation background; and 6 landraces of which (Burieuraozu, Paotsupagaiahon, Tangengenrankatsu, Nabohai, Unknown 1 and Sinceyauo), were admixtures with 44–83% of subpopulation PopIII genetic background. The Taiwanese upland *japonica* landrace, Tangengenrankatsu, containing some *indica* genetic background, was particularly interesting.

**Table 1:** The accessions of five subpopulations grouped by STRUCTURE analysis

Subpopulation	Number	Accession^**a**^
PopI	20	Tai Sen 2 (IC), Tainung Sen 19 (IC), Taichung Sen Glutinous 2 (IC), Taichung Sen 10 (IC), Tai Sen 1 (IC), Tainung Sen 20 (IC), Taichung Sen 17 (IC), Tainung Sen 12 (IC), IR 64 (IC), Taichung Native 1 (IC), Tainung Sen 14 (IC), Chianung Sen 6 (IC), Taichung Sen Glutinous 1 (IC), Kasakasth (IC), Tainung Sen 21 (IC), Tainung Sen 18 (IC), Kaohsiung Sen 7 (IC), Basmati 370 (IC), CNY922401 (IC), Tainung Sen 22(IC).
PopII	25	Ch’ien-Nung .55 (JL), Chin-Se-No (JL), Chuan .2 (JL), Chuan .4 (JL), Gurusu (JL), Kabofu (JL), Kuroca (JL), Mandarakiku (JL), Midon (JL), Munagurusu (JL), Muteka (JL), Nakarofukarapai S1 (JL), Nohrin .1 (JL), Nohrin .9 (JL), Nutsurikui (JL), Pairauwar (JL), Papito (JL), Pazumataharu (JL), Purahaitairin (JL), Ragasu (JL), Sinceyuaoho (JL), Taitungyu 46 (JL), Taitungyu 48, (JL), Taitungyu 49 (JL), Yen-No (JL).
PopIII	36	Hualien 21 (JC), Kaohsiung 139 (JC), Kaohsiung 145 (JC), Kaohsiung 146 (JC), Nipponbare (JC), Tai Keng 14 (JC), Tai Keng 16 (JC), Tai Keng 2 (JC), Tai Keng 4 (JC), Tai Keng 5 (JC), Tai Keng 8 (JC), Tai Keng 9 (JC), Tai Keng Glutinous 1 (JC), Taichung 192 (JC), Taichung 193 (JC), Taichung 65 (JC), Tainan 11 (JC), Tainan Glutinous 10 (JC), Tainung 67 (JC), Tainung 69 (JC), Tainung 70 (JC), Tainung 71 (JC), Tainung 72 (JC), Tainung 74 (JC), Tainung 75 (JC), Tainung Glutinous 73 (JC), Taitung 30 (JC), Taoyuan 3 (JC), Taoyuan 4 (JC), Burieuraozu (JL), Hung-K’o-No (JL), Nabohai (JL), Paotsupagaiahon (JL), Sinceyauo (JL), Tangengenrankatsu (JL), Unknown 1 (JL).
PopIV	53	Dular (IC), Tien-Lai (IL), T’ ai-Tung-Shung-Tung-Ch’ung (IL), T’ ai-Tung-Ta-Ma-Li-Wu-Chan (IL), Unknown 3 (IL), Shang-Chi-Tsao-Tao (IL), Chi-Shih-Jih (IL), San-Pei (IL), Baridon (IL), Wu-K’o-Tsao-Tzu (IL), Lin-Mang (IL), I-Kung-Pao (IL), Pai-Chan (IL), Chu-Tzu (IL), Parahainakoru (IL), Lei-Ch’ui (IL), Ching-Yu (IL), Kao-Chueh-Wu-Chan (IL), Pai-K′ o-Tsao-Tzu (IL), Pai-K’o-P’ u-Chan (IL), Shiau-No (IL), Jao-Yao (IL), Ch’ih-K’o (IL), Chung-Chiu No (IL), Ko-Tzu (IL), Tsui-Lo-Ku (IL), Wu-K’o (IL), Hua-Lou (IL), O-Nung .3 (IL), Hopots Utaiyaru (IL), Tuan-Li-No (IL), Cheng-wu-Chan (IL), Tan-Yang-No (IL), Unknown (IL), Cheng-Ching-Yu (IL), Chang-Hsu-Ku (IL), Wu-Chan (IL), Fukutomi (IL), Tsao-Chiu-Ku (IL), Yin-Yu-Tze (IL), Hsiao-Ko-Tzu (IL), Shuang-Chiang-Tsao-1 (IL), Wu-No-Tao (IL), Hung-No (IL), Yuan-Li (IL), Kaisentetsuchitsu (IL), Napatsupai S3 (IL), Pai-K’O-Yuan-Li (IL), Lu-Tao3036 (IL), Pakaikauneku (IL), Chin-Men-Tou-Men-Hung-Mi (IL), Pai-Mi-Fen (IL), *O. rufipogon*-21 (W).
PopV	14	Basmati 385 (IC), Taichung Sen 2 (IC), Taichung Sen 3 (IC), O. nivara-2 (W), O. nivara-5 (W), O. nivara-6 (W), O. nivara-7 (W), O. rufipogon-10 (W), O. rufipogon-12 (W), O. rufipogon-15 (W), O. rufipogon-16 (W), O. rufipogon-18 (W), O. rufipogon-19 (W), O. rufipogon-20 (W).

^a^The abbreviations in brackets () next to accessions are JL for *japonica* landrace, JC for *japonica* cultivar, IL for *indica* landrace, IC for *indica* cultivar, and W for wild rice

The indica group was subdivided into three subpopulations, PopI, PopIV and PopV (Table 1, Additional file 2: Figure S3b). Twenty accessions, including 17 Taiwanese cultivars, Basmati 370, Kasalasth, and IR64, were grouped in PopI. Four accessions (Tainung Sen 22, Kaohsiung Sen 7, Taichung Sen Glutinous 2, and Basmati 370) admixed with PopIV were noted. Most of the 53 accessions of PopIV, except Dular and *O. rufipogon*-21, were *indica* landraces. Two Taiwanese landraces (Pakaikauneku and Pai-Mi-Fen) and a Chinese landrace (Chin-Men-Tou-Men-Hung-Mi) were admixed with PopII. PopV contained 11 wild rice accessions and 3 *indica* cultivars (Taichung Sen 2, Taichung Sen 3, and Basmati 385).

The genetic diversity of each subpopulation was evaluated by using 4 parameters, mean allele number per locus, major allele frequency per locus, gene diversity and PIC value (Table 2). PopV, consisting of 11 wild rice and 3 *indica* cultivars, displayed the most diverse genetic background, revealed by the highest mean allele number (6.91), *Nei*’s gene diversity (0.74) and mean PIC value (0.71) but the lowest major allele frequency per locus (0.37) (Table 2). On the other hand, PopIII, consisting of 29 *japonica* cultivars and 7 *japonica* landraces, exhibited the lowest genetic diversity. The genetic diversities of these five subpopulations were PopV, wild rices > PopII, *japonica* landraces > PopIV, *indica* landraces > PopI, *indica* cultivars > PopIV, *japonica* cultivars. While it was anticipated that landraces would be generally more diverse than cultivars, it was noteworthy that the genetic diversity of *indica* cultivars was higher than that of *japonica* cultivars, while the genetic diversity of *indica* landraces was slightly lower than that of *japonica* landraces albeit more *indica* accessions were assessed (Table 2).

**Table 2:** Genetic diversity parameters of five subpopulations

Population^a^	Sample size	Mean allele number /locus	Major allele frequency/locus	*Nei’s* gene diversity	Mean PIC^b^ value
**All subpopulations**	148	12.71	0.36	0.75	0.72
PopI (IC)	20	4.16	0.62	0.49	0.45
PopII (JL)	25	5.89	0.50	0.60	0.57
PopIII (JC)	36	4.64	0.65	0.47	0.43
PopIV (IL)	53	6.68	0.53	0.58	0.54
PopV (W)	14	6.91	0.37	0.74	0.71

^a^The majority of accessions in PopI, PopII, PopIII, PopIV and PopV are *indica* cultivar, *japonica* landrace, *japonica* cultivar, *indica* landrace, and wild rice, respectively

^b^PIC is the abbreviation for polymorphism information content

### Genetic Divergence in Asian Cultivated *O. sativa*

The genetic diversity of the 136 *O. sativa* accessions evaluated was relatively high as revealed by mean allele number (11.01), mean gene diversity (0.74) and mean PIC (0.70) (Table 3). The highly diverse 83 landraces contributed the majority of genetic variation in this panel. Genetic diversity, in general, was higher in the *indica* than the *japonica* group; however, *japonica* landraces exhibited higher variation than *indica* landraces. The genetic diversity of cultivars was relatively narrow as compared to landraces, and *japonica* cultivars had the least variation (Table 3).

**Table 3:** Genetic diversity and divergence in *O. sativa*

Group	Number	Mean allele number/locus	Major allele frequency/locus	Mean gene diversity	Mean PIC^a^ value	*F*_ST_^b^
**All** ***O. sativa***	**136**	**11.01**	**0.36**	**0.74**	**0.70**	**0.3084**
*Indica*	75	8.31	0.50	0.61	0.58	
*Japonica*	61	7.05	0.54	0.59	0.55	
**All landraces**	**83**	**9.57**	**0.39**	**0.72**	**0.69**	**0.3040**
*Indica*	51	6.52	0.53	0.58	0.54	
*Japonica*	32	6.25	0.50	0.60	0.57	
**All cultivars**	**53**	**7.28**	**0.44**	**0.68**	**0.64**	**0.4200**
*Indica*	24	5.43	0.57	0.56	0.52	
*Japonica*	29	3.65	0.68	0.43	0.39	
**All** ***indica***	**75**	**8.31**	**0.50**	**0.61**	**0.58**	**0.1166**
Cultivars	24	5.43	0.57	0.56	0.52	
Landraces	51	6.52	0.53	0.58	0.54	
**All** ***japonica***	**61**	**7.05**	**0.54**	**0.59**	**0.55**	**0.1913**
Cultivars	29	3.65	0.68	0.43	0.39	
Landraces	32	6.25	0.50	0.60	0.57	

^a^PIC is the abbreviation for polymorphism information content

^b^Fixation index (*F*_ST_) indicates genetic differentiation between two subpopulations by the reduction of heterozygosity due to genetic drift and / or selection

Relatively high *F*_ST_ values of 0.3084 and 0.3040 was observed between *indica* and *japonica* in all *O. sativa* accessions and in landraces, respectively (Table 3). In modern breeding under intensified directional selection, *indica* and *japonica* cultivars are even more diversified from each other as revealed by the highest *F*_ST_ value (0.4200). On the other hand, there was less divergence between cultivars and landraces both in *indica* and *japonica*.

### Divergence of Taiwanese Rice Germplasm

In the collection of 106 Taiwanese accessions, the genetic diversity of *indica* accessions was higher than that of *japonica* ones, and the genetic diversity of landraces was also higher than cultivars (Table 4). The genetic diversity of *indica* landraces was not obviously different from *japonica* landraces; however, *indica* cultivars exhibited greater diversification than *japonica* cultivars. Taiwanese cultivars were divided into ‘early’ cultivars or ‘late’ cultivars consistent with the government policy of rice breeding goals changing from yield (early) to premium grain quality (late) in 1981. The 25 late *japonica* cultivars exhibited larger genetic diversity than 3 early *japonica* cultivars; nevertheless, the difference was not statistically significant difference between early and late *indica* cultivars (Table 4).

**Table 4:** Genetic diversity and divergence in Taiwanese germplasm

Group	Number	Mean allele number/locus	Major allele frequency/locus	Mean gene diversity	Mean PIC value	*F*_ST_
**All Taiwanese accessions**	**106**	**10.19**	**0.36**	**0.74**	**0.70**	**0.3181**
*Indica*	55	7.41	0.50	0.61	0.57	
*Japonica*	51	6.43	0.56	0.57	0.54	
**All Taiwanese landraces**	**59**	**8.55**	**0.39**	**0.72**	**0.68**	**0.3142**
*Indica*	36	5.73	0.53	0.58	0.53	
*Japonica*	23	5.37	0.52	0.58	0.55	
**All Taiwanese cultivars**	**47**	**6.84**	**0.46**	**0.66**	**0.62**	**0.4251**
*Indica*	19	4.81	0.58	0.54	0.51	
*Japonica*	28	3.63	0.68	0.42	0.38	
**Taiwanese** ***indica*** **accessions**	**55**	**7.41**	**0.50**	**0.61**	**0.57**	**0.1344**
Cultivars	19	4.81	0.58	0.54	0.51	
Landraces	36	5.73	0.53	0.58	0.53	
**Taiwanese** ***japonica*** **accessions**	**51**	**6.43**	**0.56**	**0.57**	**0.54**	**0.1995**
Cultivars	28	3.63	0.68	0.42	0.38	
Landraces	23	5.37	0.52	0.58	0.55	
**All Taiwanese** ***indica*** **cultivars**	**19**	**4.81**	**0.58**	**0.54**	**0.51**	**0.0045**
Early cultivars^a^	7	3.29	0.59	0.52	0.47	
Late cultivars	12	3.93	0.61	0.51	0.47	
**All Taiwanese** ***japonica*** **cultivars**	**28**	**3.63**	**0.68**	**0.42**	**0.38**	**0.3751**
Early cultivars	3	1.72	0.76	0.29	0.24	
Late cultivars	25	3.47	0.68	0.42	0.38	

^a^The early and late cultivars were released before and after 1981, respectively

For the Taiwanese accessions, great differentiation between *indica* and *japonica* types was indicated by high *F*_ST_ (0.3181), with Taiwanese landraces similar to this overall trend (*F*_ST_ = 0.3142) but higher differentiation between *indica* and *japonica* cultivars (*F*_ST_ = 0.4251) (Table 4). Less differentiation between Taiwanese *japonica* cultivars and landraces (*F*_ST_ = 0.1995) and *indica* cultivars and landraces (*F*_ST_ = 0.1344) were observed. The late *indica* cultivars were not differentiated from the early *indica* cultivars (*F*_ST_ = 0.0045); however, the late *japonica* cultivars were significantly differentiated from the early *japonica* cultivars (*F*_ST_ = 0.3751).

### Relatedness Based on Genetic Distances

**Fig. 1:**
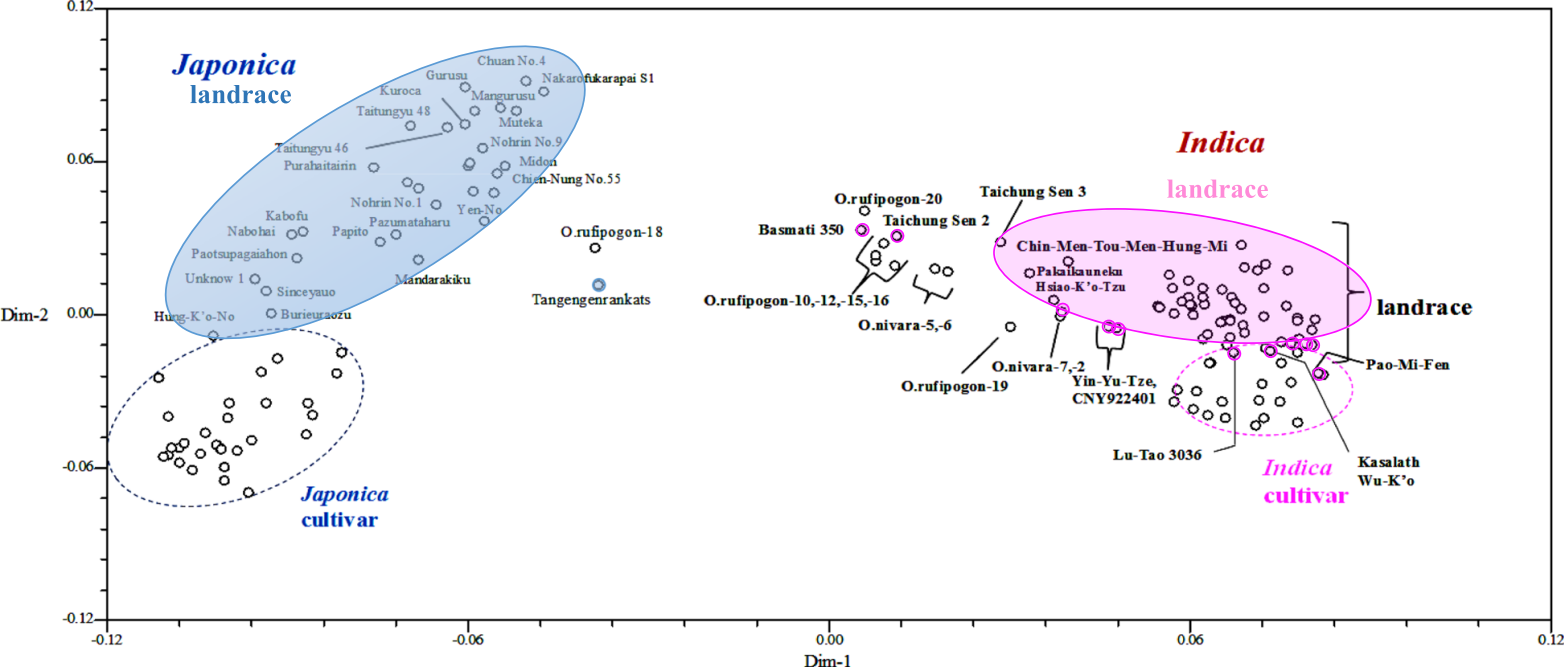
Principle coordinate analysis (PCoA) of 148 rice accessions. After 2-dimension analysis of PCoA, the first and second dimension explained 18.1% and 7.7%, respectively. Each accession is indicated by a circle. *Japonica* and *indica* rices are distinctly separated, with *japonica* and *indica* landraces indicated by blue and magenta solid ellipses, and *japonica* and *indica* cultivars indicated by blue and  magenta dashed ellipses, respectively

The 148 accessions could be separated into two groups corresponding to *indica* and *japonica* by 2-dimensional PCoA analysis, in which the first and the second dimension explained 18.1% and 7.7% of variation, respectively (Fig. 1). *Japonica* accessions were distinct from *indica* accessions, and *japonica* accessions were distributed more sparsely than *indica* accessions. The *indica* cultivars could be distinguished from *indica* landraces by the third dimension, accounting for 3.12% of variation (Additional file 2: Figure S4). The cultivars were more closely aggregated than landraces for *indica* and *japonica*, indicating more similar genetic background. Three landraces (Tangengenrankatsu, Pakaikauneku and Hsiao-K’o-Tzu) and 2 cultivars (Taichung Sen 2 and Taichung Sen 3) were close to wild rice.

**Fig. 2:**
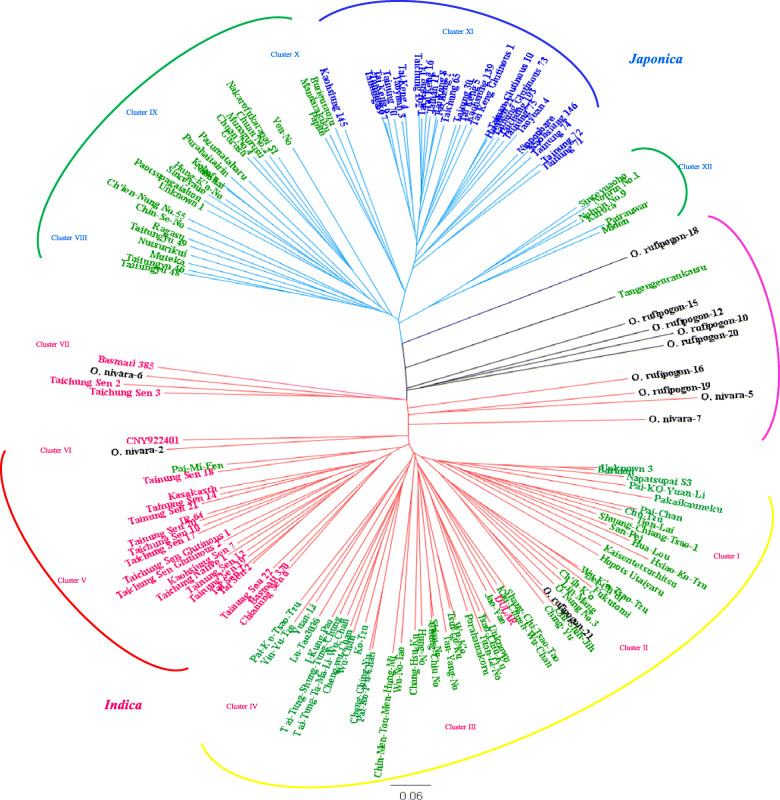
Unrooted neighbor-joining tree of 148 rice accessions. Genetic distance was calculated according to Nei (1983) with the genotypes of 75 markers and cluster analysis by the neighbor-joining method. *Japonica* cultivars, *indica* cultivars, landrace and wild rices are indicated by blue, red, green and black, respectively. Cluster I-VII belong to indica sub-groups and Cluster VIII-XII belong to japonica sub-groups. Bar represents genetic distance

The unrooted phylogenetic tree according to *Nei’s* genetic distances revealed two distinct major clusters, indica and japonica, indicated by red and blue branches, respectively (Fig. 2). The indica cluster could be further subdivided into 7 clades, Clade I to VII, and the japonica cluster could be subdivided into 5 clades, Clade VIII to XII. The *indica* cultivars were grouped in Clade V and distinguished from the *indica* landraces, which formed one group with 4 distinct clades, Clade I – IV. Clades VIII, IX, X and XII were primarily comprised of *japonica* landraces. On the other hand, *japonica* cultivars entirely constituted Clade XI, with the exception that Kaohsiung 145 was closely-related to three *japonica* landraces, Burieuraozu, Mandarakiku and Papito. These results show that the genetic background of modern *indica* and *japonica* cultivars have deviated from those of traditional landraces under intensive selection for breeding goals of high yield and premium grain quality. Finally, wild rice, indicated with a black branch, could not form a distinct group and fell into intermediate locations in the phylogenetic tree. One *japonica* landrace, Tangengenrankatsu, and four *indica* cultivars (Basmati 385, Taichung Sen 2, Taichung Sen 3, and CNY922401) also fell into intermediate locations allied with wild rice.

## Discussion

### Genetic Diversity Revealed by Assessment of Molecular Markers

Genetic diversity evaluated by molecular markers provides useful and fundamental information for crop improvement. Among an assortment of markers, SSRs exhibiting relatively high polymorphism level per locus provide rich allelic information for genetic diversity analysis. A currently-preferred phylogenetic relationship of rice germplasm was established by using SSRs that discerned 5 major groups – specifically, *aus*, *indica*, *aromatic*, *temperate japonica* and *tropical japonica* (Garris et al. 2005). Phylogenetic trees of rice germplasm using genome-wide SNPs or structural variants were in accordance with these 5 groups (Wang et al. 2018; McNally et al. 2009; Fuentes et al. 2019). In the present study, a total of 953 alleles were detected by 75 polymorphic markers, varying from 3 to 37 alleles per locus with an average of 12.7 (Additional file 2: Figure S1, Additional file 1: Table S2), which was higher than other studies (Chakhonkaen et al. 2012; Jin et al. 2010; Nachimuthu et al. 2015). PIC values, which are good indicators of marker polymorphism levels, were in the range of 0.18 to 0.95, with a mean of 0.72, higher than those reported in European (0.49), Chinese (0.42) and Indonesian (0.66) rice germplasm, respectively (Courtois et al. 2013; Jin et al. 2010; Thomson et al. 2007). Moreover, 66 of 75 markers (88%) were considered highly informative with PIC values > 0.5 (Additional file 2: Figure S1). Thus, these 75 markers provided rich allelic information for genetic diversity analysis.

### The Genetic Diversity and Differentiation in the Collected Rice Germplasm

The diversity panel of 148 accessions could be separated into two subpopulations according to STRUCTURE analysis, clearly corresponding to *indica* and *japonica* groups (Additional file 2: Figure S3a). Further division into five subpopulations, *indica* cultivars, *indica* landraces, *japonica* cultivars, *japonica* landraces and wild rice, were supported by *K* = 5 (Table 1, Additional file 2: Figure S3b). Most accessions were classified into the expected groups according to the records of the National Plant Genetic Resource Center (NPGRC) Taiwan that were classified by plant and seed morphology, however some were incongruent due to admixed genetic background. For example, landraces, Sinceyauo from Japan, Hung-K’o-No from China and Burieuraozu, Nabohai, Paotsupagaiahon, and Tangengenrankatsu from Taiwan, were grouped with *japonica* cultivars (Pop III); an aus cultivar (Dular) and *O. rufipogon*-21 were assigned to the *indica* landrace group (Popn IV); and three *indica* cultivars, Basmati 385, Taichung Sen 2 and Taichung Sen 3, were placed in the wild rice group (PopV) (Table 1). These accessions might still share identical by descent segments since derivation from common ancestors. One possible factor contributing to such incongruous findings that cannot be neglected is introgression owing to gene flow among wild species, landraces, and cultivars (Ishikawa et al. 2006; Wang et al. 2018). For example, a mega variety TC65 inherited photoperiod-insensitive alleles of *Heading date 1* (*Hd1*) and *Early heading date 1* (*Ehd1*) from two landraces, Muteke and Nakabo, by spontaneous introgression of natural gene flow during modern breeding (Wei et al. 2016). Indeed, landraces have been commonly used in breeding programs especially in the early purification breeding stage e.g. two old Taiwanese *indica* cultivars, Taichung Sen 2 and Taichung Sen 3, derived from landraces based on breeding records. Thus, admixed accessions are not necessarily rare outcomes of natural introgression, but derive from intentional cross hybridization in at least some cases.

Morphology and genetic background are quite different between *indica* and *japonica* rice through independent origins, long-term adaptation to diverse environments and selection for various human preferences. The extent of genetic differentiation between these two subspecies was revealed by *F*_ST_ analysis (Ikehashi 2009; Zhang et al. 2007). High genetic differentiation (*F*_ST_ = 0.3084) was observed between *indica* and *japonica* groups in our rice diversity panel (Table 3), in agreement with several studies (Thomson et al. 2007; Lin et al. 2012). The level of differentiation between *indica* and *japonica* landraces (*F*_ST_ = 0.3040) was lower than that between *indica* and *japonica* cultivars (*F*_ST_ = 0.4200). Landraces were selected by farmers for adaptation to local environments and various preferences; while modern cultivars result from intense directional selection for specific traits. Less differentiation in landraces than in cultivars was associated with different selection intensity.

The gene diversity of *indica* accessions was higher than that of *japonica* accessions since the bottleneck effect was more severe in *japonica* rice during early domestication (Kovach and McCouch 2008; Wang et al. 2018; Zhu et al. 2007). In the present study, genetic diversity was much lower in *japonica* than *indica* populations as well (Fig. 1, Table 3), the same tendency as in previous studies using Taiwan breeding germplasm and a collection from Borneo Island (Lin et al. 2012; Thomson et al. 2009). Nevertheless, in our collection the level of diversity of *japonica* landraces was higher than that of *indica* landraces (Table 3) because the former included both upland and lowland accessions.

### Unveiling Taiwanese Rice Germplasm

Today, indigenous peoples still cultivate their own landraces with unique traits, such as large grain and aroma, on upland fields in Taiwan. The cultivation of rice, accompanied by foxtail millet, can be dated back to 5000 years ago by unearthed grain remains from some archaeological sites in Tainan Science Park, southern Taiwan (Tsang 2012). Approximately 98% and 83% of the excavated carbonized rice grains from the Tapenkeng Culture period (4800–4200 B.P.) and Niuchoutzu Culture period (3800–3300 B.P), respectively, were classified as *japonica* rice according to grain morphology (Tsang 2012; Wang 2007).

In the present study, 17 landraces labeled with ‘^#^’ in the Additional file 1: Table S1, were grouped in Clusters VIII, IX, X and XII which belong to the *japonica* clade (Fig. 2). These indigenous landraces were genetically distinct from modern temperate *japonica* cultivars, Cluster XI (Fig. 2), and presumed to belong to *tropical japonica* rice (*javanica*). The upland landrace, Tangengenrankatsu, has admixed genetic background and is genetically close to *O. rufipogon*-18. Only few indigenous landraces were clustered in *indica* clades, albeit some were classified as *japonica* rice by morphology according to NPGRC records, such as Pakaikauneku, Kaisentetsuchitsu, Napatsupa S3, and Baridon (Additional file 1: Table S1, Fig. 2). *Tropical japonica*, diverged from *temperate japonica*, is thought to have originated in the upper Thai-Malay Peninsula and might have moved from the Malay Archipelago northward through Indonesia, the Philippines, Taiwan, Ryukyus, and Japan (Chang 1976; Gutaker et al. 2020). Thus, Taiwan was on the dispersal route of *tropical japonica* and 2/3 of carbonized rice grains unearthed from remains of Niaosung Culture (1400–1000 B.P.) had grain length larger than 4 mm which resembled *tropical japonica* (Wang 2007). In accordance with *archaeobotanical* evidence, phylogenetic analysis of SSR genotypes classified indigenous upland landraces as *tropical japonica* (Fig. 2).

In the *indica* clusters, only 6 accessions were recorded with indigenous language pronunciations, including 5 (Baridon, Napatsupai S3, Pakaikameku, Kaisentetsuchitsu, Hopot Utatyaru) in cluster I and Parahainakoru in cluster III (Fig. 2). These 6 *indica* landraces might have been preserved and cultivated by indigenous people for thousands of years, however archaeobotanical evidence is lacking. We cannot rule out that these *indica* landraces were adopted by indigenous people only hundreds of years ago, after Chinese introduced much *indica* rice. The landraces in cluster II – IV came from Taiwan and China and showed no significant isolation-by-distance (Fig. 2). However, the *indica* landraces were divided into two large clades, Cluster I & II and Cluster III & IV, which might reflect two origins, Guangdong and Fujian. The genetic diversity of *indica* landraces in Taiwan is relatively high (Tables 3, 4) which might result from intrinsic high variation in *indica* rice and multiple origins as well. Taiwanese *indica* cultivars, closer to IR64 than Dular, an Aus cultivar in India (Fig. 2), might be resulted from modern breeding that 14 of 17 Taiwanese *indica* cultivars can be traced back to IRRI accessions or DGWG as their breeding parents (Lu and Lu 2010).

Landraces, intermediate between wild relatives and cultivars, are important genetic reservoir for crop improvement to cope with climate changes and increase sustainability. In Taiwan, 16 officially acknowledged indigenous peoples have their own cultures and diet preferences, including diversified crop germplasm. Taiwanese rice landraces compromised of *tropical japonica* and *indica* rice revealed diverse genetic variation in plant architecture and seeds (Hsieh et al. 2011) and herein showed much SSR diversity (Tables 3, 4). This high genetic variation indicates that Taiwanese landraces are a reservoir of genetic diversity and beneficial genes/alleles for rice breeding and improvement. Taiwanese landraces have had great impact on modern rice breeding not only in Taiwan but also elsewhere in the world. According to the database of rice breeding pedigrees (Taiwan Rice Information System, TRIS), Taiwanese landraces were commonly used to introgress useful genes for rice improvement, especially in the early breeding programs a half-century ago. The most prominent varieties, *japonica* TC65 with photoperiod insensitivity and *indica* variety Taichung Native 1 (TCN1) with semi-dwarf stature, have had great impact on rice breeding and research. Because photoperiod insensitivity was a highly desired trait, TC65 had been extensively applied in modern rice breeding programs, leading to all current Taiwanese *temperate japonica* cultivars inheriting the *ehd1* and *hd1* alleles. Taiwanese *temperate japonica* cultivars can be cultivated in two crop seasons under tropical and subtropical environments, making Taiwan the southernmost region of *temperate japonica* cultivation. The *indica* variety TCN1 inherited null function of *sd1* with a 383-bp deletion from the landrace DGWG (Sasaki et al. 2002), and, this DGWG allele has been widely applied to improve grain yield of both *indica* and *japonica* varieties in the past 50 years (Asano et al. 2011; Zhao et al. 2010). Yet, there are still numerous useful genes/alleles existing in the genetic reservoir of Taiwanese landraces, for example conferring large grain size, aroma, and biotic and abiotic resistance. Untapped beneficial genes from landraces can help to breed new varieties for resilient and sustainable agriculture.

Modern cultivars are a result of intensive directional selection for specific traits which are frequently determined by government policy and demands of markets. In Taiwan, the major dining staple was *indica* rice before War World II but changed to *temperate japonica* rice because of government policy during Japanese occupation. Now, *japonica* rice is for dining; while *indica* rice is used for various food processing needs, such rice noodles, pudding, and cakes. Thus, *japonica* and *indica* improvement have different breeding goals. For *indica* rice, high yield with resistances to biotic and abiotic stresses are breeding goals; thus, diverse germplasm from landraces or introduced from other countries are commonly utilized as donor parents (Lin et al. 2012). Therefore, there was no obvious difference in genetic diversity and differentiation between early and late *indica* cultivars (*F*_ST_ = 0.0045, Table 4). On the other hand, the breeding goal of *japonica* rice was changed from high yield to premium grain quality that the germplasm used for improving different traits seemed to be associated with high differentiation between early and late *japonica* cultivars, *F*_ST_ = 0.3751 (Table 4). In order to improve grain quality, a few Japanese elite *temperate japonica* cultivars were introduced and used extensively in recurrent breeding crosses (Lin et al. 2012). This led to modern Taiwanese *japonica* cultivars being grouped at the same clade, Cluster XI with the Japanese elite cultivar, Nipponbare (Fig. 2), as *japonica* varieties from Taiwan and Japan did not differ significantly in the pattern of genetic diversity (Lin et al. 2012). The genetic distances between any two Taiwanese *japonica* cultivars were in the range of 0.43–0.58 (Fig. 2); consequently, the gene pool of *japonica* cultivars is relative narrow as compared to either *japonica* landraces or modern *indica* cultivars (Tables 3, 4), resulting in genetic vulnerability in rice cultivation and management.

To overcome severe genetic vulnerability of *temperate japonica* cultivars, wild relatives and *indica* rice were introduced to breeding programs. For example, *japonica* Tainung 67 was the descent of a cross of *japonica* Tainung 61 and *O. rufipogon*, and *japonica* Taichung 192 was an *indica*/*japonica*-crossed variety (Lu and Lu 2010). Recently, numerous advanced breeding lines introduced from IRRI and wild relatives have been used in breeding programs to improve biotic and abiotic stresses for sustainable agriculture, e. g. IRBB66 pyramided with 5 bacterial blight resistant genes (Yap et al. 2016). Thus, current rice breeding goals in Taiwan emphasize grain quality first, followed by other traits such as resistances and multi-dimensional utilizations (forage and landscape). To achieve various goals, germplasm for breeding are not limited to the domestics but also exotics.

## Conclusions

A diversity panel of 148 rice accessions, including 47 cultivars and 59 landraces from Taiwan and 42 accessions from other countries, could be grouped into five major subpopulations: wild rices, *japonica* landraces, *indica* landraces, *indica* cultivars, and *japonica* cultivars. The genetic diversities, without exception, were wild rices > landraces > cultivars, and *indica* rice > *japonica* rice. The majority of Taiwanese *japonica* landraces preserved by indigenous peoples were classified as *tropical japonica* by morphology and phylogenetic analysis, consistent with *archaeobotanical* evidence. Thus, *japonica* landraces had greater genetic variation than *indica* landraces. The Taiwanese *indica* landraces could be separated into two clusters on phylogenetic trees, reflecting two sets of introductions from China. The genetic variation and divergence of modern cultivars are largely influenced by government policies and market demands, exemplified by premium grain quality for *japonica* rice, and yield and resistances for *indica* rice. Large genetic diversification was unveiled in Taiwanese landraces, as well as intermediate and admixed genetic background, providing a precious and valuable genetic reservoir for rice breeding in the future.

## Materials and Methods

### Plant Materials

A diversity panel of 148 rice accessions, including 136 *O. sativa*, 4 *O. nivara*, and 8 *O. rufipogon*, were analyzed in this study. The germplasm originated from Taiwan, Japan, and China or was introduced from the International Rice Research Institute (IRRI), and was obtained from the National Plant Genetic Resource Center (NPGRC), Taiwan. These germplasms were propagated and used for rice improvement by rice breeders, Dr. Chih-Shen Sheu in Taichung District Agricultural Research and Extension Station and Dr. Yong-pei Wu in Chiayi Agricultural Experiment Branch, Taiwan Agricultural Research Institute. Each accession denoted *indica* or *japonica* and cultivars or landraces was in accordance with the record in NPGRC based on the classification according to morphology and collection sites. For cultivars, there were 29 *japonica* varieties including 28 Taiwanese and 1 Japanese cultivar and 24 *indica* varieties including 19 Taiwanese, 1 Pakistani and 3 Indian cultivars, and IR64. For landraces, there were 59, 18, and 6 accessions from Taiwan, China and Japan, respectively. For the 59 Taiwanese landraces, 23 accessions were recorded with pronunciations of indigenous languages, 36 with Chinese characters, and 3 labeled as Unknown, Unknown 1 and Unknown 3, respectively. The 12 wild rices were collected from China, Bangladesh and Laos (Additional file 1: Table S1).

### DNA Extraction and Genotyping Assay

Genomic DNA was extracted from leaf tissues of rice seedlings at the three-leaf stage as described previously (Lin et al. 2012). A total of 75 markers including 49 published SSRs (McCouch et al. 2002), 6 STSs (Wu et al. 2010), and 20 newly-designed SSRs and indels (Additional file 1: Table 2) distributed across the rice genome were applied for genotyping assay.

Among the 75 markers, 56 were analyzed with a QIAxcl System -GT12™ Genetic Analyzer (Qiagen, USA). The PCR reaction was in a total volume of 15 μL containing 30 ng genomic DNA, 0.3 nmol/μL forward and reverse primer each, and 8 μL T*aq* DNA Polymerase Master Mix (Ampliqon, Denmark). Amplification was performed on a thermocycler (Model T1, Biometra, Germany) with the following thermal profile: 94 °C for 3 min for 1 cycle; 94 °C for 40 s, 55 °C for 40 s, 72 °C for 40 s, for 35 cycles; 72 °C for 3 min for 1 cycle. Amplicons were resolved by QIAxcel DNA High Resolution Kit (1200) with QX size marker 25–450 and QX alignment marker 15 bp/500 bp (Qiagen, USA). The other 19 markers were assessed on an ABI 3730 DNA Analyzer (Applied BioSystems, USA). PCR reactions were set in a total volume of 20 μL containing 20 ng of genomic DNA, 10 pmol/μL of primer labeled with a fluorescent dye, 2 μL of 10× PCR buffer, 2 μL of 2.5 nmol/μL dNTPs, 1.5 μL of 5 U/μL Amplitaq Gold® DNA polymerase (Applied Biosystems, USA), and 2 μL of 1 mol/L betaine. Amplifications were performed with the following thermal profile: 94 °C for 5 min for 1 cycle; 95 °C for 30 s, 55 °C for 55 s, 72 °C for 35 s, for 35 cycles; and 72 °C for 1 min for 1 cycle. DNA fragment analysis of amplified products were carried out by using an ABI 3730 DNA Analyzer with ABI GeneScan™ -600 LIZ™ Size Standard following the manufacturer’s instructions (Applied BioSystems, USA).

### Data Analysis

To evaluate genetic relatedness of these 148 accessions, genotypes of 75 markers were subjected to genetic diversity, population structure simulation, principle coordinate analysis (PCoA), and phylogenetic analysis. Five genetic diversity parameters including mean allele number per locus, major allele frequency per locus, *Nei’s* gene diversity, mean polymorphic information content (PIC), and fixation index (*F*_ST_) were assessed by using PowerMarker V3.25 (Liu and Muse 2005).

Population structures of 148 accessions were analyzed by STRUCTURE V 2.3.3, a Bayesian model-based approach (Pritchard et al. 2000). Simulation was performed under the admixture model with 100,000 burn-in iterations of Markov Chain Monte Carlo (MCMC) for *K* values set from 1 to 11, and *∆K* (an ad hoc quantity) was used to determine subpopulation number (Evanno et al. 2005).

The genetic distance of similarity matrix was calculated using modified Rogers’ distance (Goodman and Stuber 1983). The genetic distances were consequently subjected to two-dimension principle coordinate analysis with Decnter and Eigene modules (Rohlf 1987) and used in construction of an unrooted phylogenetic tree by neighbor-joining in PowerMarker V3.25 (Liu and Muse 2005) and visualized using TreeView.

## Additional Files

**Additional file 1: Table S1.** Name, type, subspecies/species and origin of 148 rice accessions used in this study. **Table S2.** Chromosomal position, locus name and PIC value of 75 SSR marker used for this study.

**Additional file 2: Figure S1.** The frequency distribution of allele number and polymorphic information content (PIC) with 75 molecular markers. (A) Allele number per locus ranges from 3 to 37 with an average of 12.7. (B) PIC ranges from 0.18 to 0.95 with an average of 0.72. **Figure S2.** (a) Structure simulation analysis to determine best *K*. (A) LnP(D), the log likelihood for each *K*, was calculated by 100,000 permutations and mean LnP(D) value was taken from 10 replications. *∆K*, an ad hoc quantity, is transferred by mean LnP(D) value and *∆K* of 148 accessions. (B) LnP(D) value and *ΔK* of 86 *indica* accessions. (C) LnP(D) value and *ΔK* of 86 *japonica* accessions. **Figure S3.** Population structure analysis of 148 accessions. Each individual is indicated by a vertical bar. (A) For *K* = 2, pop2–1 and pop2–2, indicated by red and green, are composed of *japonica* and *indica* rice, respectively. (B) For *K* = 5, pop5–1, pop5–2, pop5–3, pop5–4 and pop5–5, indicated by red, green, blue, yellow and magenta, are composed of *indica* cultivar, *japonica* landrace, *japonica* cultivar, *indica* landraces and wild rices, respectively. The numbers of accessions in each subpopulation are indicated in brackets (). **Figure S4.** Three-dimensional plot from principle coordinate analysis of 148 rice accessions. *Japonica* and *indica* are separated on opposite sides. *Japonica* and *indica* cultivars are marked with circles.

## Data Availability

Dataset and figures supporting the results are included as additional files.

## Abbreviations

IRRI: International Rice Research Institute
SNP: Single nucleotide polymorphism
DGWG: Dee-Geo-Woo-Gen
PCoA: Principal coordinate analysis
SSR: Simple sequence repeat
STS: Sequence-tagged site
indel: Insertion/deletion
PIC: Polymorphic information content
Pop: Population
NPGRC: National Plant Genetic Resource Center
TC65: Taichung 65
TCN1: Taichung Native 1
