# Supplementary material for: Genetic Diversity of Landraces and Improved Varieties of Rice (Oryza sativa L.) in Taiwan
Source: Rice (N Y). 2020 Dec 14;13:82. doi: 10.1186/s12284-020-00445-w (PMC7736384; doi:10.1186/s12284-020-00445-w)
Supplement: Supplementary file 1 — Additional file 1: Table S1. Name, type, subspecies/species and origin of 148 rice accessions used in this study. Table S2. Chromosomal position, locus name and PIC value of 75 SSR marker used for this study. [file 12284_2020_445_MOESM1_ESM.docx]

**Additional file 1: Table S1. Name, type, subspecies/species type and origin of 148 rice accessions used in this study. (Continued)**

| Name | Type | Subspeices/species | Origin |
| --- | --- | --- | --- |
| Chianung Sen 6 | cultivar | indica | Taiwan |
| CNY922401 | cultivar | indica | Taiwan |
| Kaohsiung Sen 7 | cultivar | indica | Taiwan |
| Tai Sen 1 | cultivar | indica | Taiwan |
| Tai Sen 2 | cultivar | indica | Taiwan |
| Taichung Native 1 | cultivar | indica | Taiwan |
| Taichung Sen Glutinous 1 | cultivar | indica | Taiwan |
| Taichung Sen Glutinous 2 | cultivar | indica | Taiwan |
| Taichung Sen 10 | cultivar | indica | Taiwan |
| Taichung Sen 17 | cultivar | indica | Taiwan |
| Taichung Sen 2 | cultivar | indica | Taiwan |
| Taichung Sen 3 | cultivar | indica | Taiwan |
| Tainung Sen 12 | cultivar | indica | Taiwan |
| Tainung Sen 14 | cultivar | indica | Taiwan |
| Tainung Sen 18 | cultivar | indica | Taiwan |
| Tainung Sen 19 | cultivar | indica | Taiwan |
| Tainung Sen 20 | cultivar | indica | Taiwan |
| Tainung Sen 21 | cultivar | indica | Taiwan |
| Tainung Sen 22 | cultivar | indica | Taiwan |
| Basmati 370 | cultivar | indica | India |
| DULAR | cultivar | indica | India |
| Kasakasth | cultivar | indica | India |
| Basmati 385 | cultivar | indica | Pakistan |
| IR 64 | cultivar | indica | IRRI |
| Hualien. 21 | cultivar | japonica | Taiwan |
| Kaohsiung 139 | cultivar | japonica | Taiwan |
| Kaohsiung 145 | cultivar | japonica | Taiwan |
| Kaohsiung 146 | cultivar | japonica | Taiwan |
| Tai Keng Glutinous 1 | cultivar | japonica | Taiwan |
| Tai Keng 14 | cultivar | japonica | Taiwan |

**Additional file 1: Table S1. Name, type, subspecies/species and origin of 148 rice accessions used in this study. (Continued)**

| Name | Type | Subspeices/species | Origin |
| --- | --- | --- | --- |
| Tai Keng 2 | cultivar | japonica | Taiwan |
| Tai Keng 4 | cultivar | japonica | Taiwan |
| Tai Keng 5 | cultivar | japonica | Taiwan |
| Tai Keng 8 | cultivar | japonica | Taiwan |
| Tai Keng 9 | cultivar | japonica | Taiwan |
| Tai Keng 16 | cultivar | japonica | Taiwan |
| Taichung 192 | cultivar | japonica | Taiwan |
| Taichung 193 | cultivar | japonica | Taiwan |
| Taichung 65 | cultivar | japonica | Taiwan |
| Tainan Glutinous 10 | cultivar | japonica | Taiwan |
| Tainan 11 | cultivar | japonica | Taiwan |
| Tainung Glutinous 73 | cultivar | japonica | Taiwan |
| Tainung 67 | cultivar | japonica | Taiwan |
| Tainung 69 | cultivar | japonica | Taiwan |
| Tainung 70 | cultivar | japonica | Taiwan |
| Tainung 71 | cultivar | japonica | Taiwan |
| Tainung 72 | cultivar | japonica | Taiwan |
| Tainung 74 | cultivar | japonica | Taiwan |
| Tainung 75 | cultivar | japonica | Taiwan |
| Taitung 30 | cultivar | japonica | Taiwan |
| Taoyuan 3 | cultivar | japonica | Taiwan |
| Taoyuan 4 | cultivar | japonica | Taiwan |
| Nipponbare | cultivar | japonica | Japan |
| Baridon | landrace^#^ | indica | Taiwan |
| Chu-Tzu | landrace | indica | Taiwan |
| Cheng-Ching-Yu | landrace | indica | Taiwan |
| Cheng-wu-Chan | landrace | indica | Taiwan |
| Ch'ih-K'o | landrace | indica | Taiwan |
| Ching-Yu | landrace | indica | Taiwan |
| Chi-Shih-Jih | landrace | indica | Taiwan |

**Additional file 1: Table S1. Name, type, subspecies/species and origin of 148 rice accessions used in this study. (Continued)**

| Name | Type | Subspeices/species | Origin |
| --- | --- | --- | --- |
| Hopots Utaiyaru | landrace^#^ | indica | Taiwan |
| Hsiao-Ko-Tzu | landrace | indica | Taiwan |
| Hua-Lou | landrace | indica | Taiwan |
| I-Kung-Pao | landrace | indica | Taiwan |
| Jao-Yao | landrace | indica | Taiwan |
| Kaisentetsuchitsu | landrace^#^ | indica | Taiwan |
| Kao-Chueh-Wu-Chan | landrace | indica | Taiwan |
| Ko-Tzu | landrace | indica | Taiwan |
| Lei-Ch'ui | landrace | indica | Taiwan |
| Lin-Mang | landrace | indica | Taiwan |
| Napatsupai S3 | landrace^#^ | indica | Taiwan |
| Pai-Chan | landrace | indica | Taiwan |
| Pai-K’o-Tsao-Tzu | landrace | indica | Taiwan |
| Pai-Ko-P’u-Chan | landrace | indica | Taiwan |
| Pai-K’O-Yuan-Li | landrace | indica | Taiwan |
| Pai-Mi-Fen | landrace | indica | Taiwan |
| Pakaikauneku | landrace^#^ | indica | Taiwan |
| Parahainakoru | landrace^#^ | indica | Taiwan |
| San-Pei | landrace | indica | Taiwan |
| Shuang-Chiang-Tsao-1 | landrace | indica | Taiwan |
| T’ai-Tung-Shung-Tung-Ch’ung | landrace | indica | Taiwan |
| T’ai-Tung-Ta-Ma-Li-Wu-Chan | landrace | indica | Taiwan |
| Tien-Lai | landrace | indica | Taiwan |
| Wu-Chan | landrace | indica | Taiwan |
| Wu-K'o | landrace | indica | Taiwan |
| Wu-K'o-Tsao-Tzu | landrace | indica | Taiwan |
| Yuan-Li | landrace | indica | Taiwan |
| Chang-Hsu-Ku | landrace | indica | China |
| Chin-Men-Tou-Men-Hung-Mi | landrace | indica | China |
| Chung-Chiu No | landrace | indica | China |

**Additional file 1: Table S1. Name, type, subspecies/species and origin of 148 rice accessions used in this study. (Continued)**

| Name | Type | Subspeices/species | Origin |
| --- | --- | --- | --- |
| Hung-No | landrace | indica | China |
| Lu-Tao 3036 | landrace | indica | China |
| O-Nung 3 | landrace | indica | China |
| Shang-Chi-Tsao-Tao | landrace | indica | China |
| Shiau-No | landrace | indica | China |
| Tan-Yang-No | landrace | indica | China |
| Tsao-Chiu-Ku | landrace | indica | China |
| Tsui-Lo-Ku | landrace | indica | China |
| Tuan-Li-No | landrace | indica | China |
| Wu-No-Tao | landrace | indica | China |
| Yin-Yu-Tze | landrace | indica | China |
| Fukutomi | landrace | indica | Japan |
| Burieuraozu | landrace^#^ | japonica | Taiwan |
| Chuan 2 | landrace | japonica | Taiwan |
| Chuan 4 | landrace | japonica | Taiwan |
| Gurusu | landrace^#^ | japonica | Taiwan |
| Kabofu | landrace^#^ | japonica | Taiwan |
| Mandarakiku | landrace^#^ | japonica | Taiwan |
| Midon | landrace^#^ | japonica | Taiwan |
| Munagurusu | landrace^#^ | japonica | Taiwan |
| Muteka | landrace^#^ | japonica | Taiwan |
| Nabohai | landrace^#^ | japonica | Taiwan |
| Nakarofukarapai S1 | landrace^#^ | japonica | Taiwan |
| Nutsurikui | landrace^#^ | japonica | Taiwan |
| Pairauwar | landrace^#^ | japonica | Taiwan |
| Paotsupagaiahon | landrace^#^ | japonica | Taiwan |
| Papito | landrace^#^ | japonica | Taiwan |
| Pazumataharu | landrace^#^ | japonica | Taiwan |
| Purahaitairin | landrace^#^ | japonica | Taiwan |
| Ragasu | landrace^#^ | japonica | Taiwan |
|  |  |  |  |

**Additional file 1: Table S1. Name, type, subspecies/species and origin of 148 rice accessions used in this study.**

| Name | Type | Subspeices/species | Origin |
| --- | --- | --- | --- |
| Taitungyu 46 | landrace | japonica | Taiwan |
| Taitungyu 48 | landrace | japonica | Taiwan |
| Taitungyu 49 | landrace | japonica | Taiwan |
| Tangengenrankatsu | landrace^#^ | japonica | Taiwan |
| Ch'ien-Nung .55 | landrace | japonica | China |
| Chin-Se-No | landrace | japonica | China |
| Hung-K'o-No | landrace | japonica | China |
| Kuroca | landrace | japonica | Japan |
| Nohrin 1 | landrace | japonica | Japan |
| Nohrin 9 | landrace | japonica | Japan |
| Sinceyauo | landrace | japonica | Japan |
| Sinceyuaoho | landrace | japonica | Japan |
| *O. nivara-2* | wild rice | nivara | Bangladesh |
| *O. nivara-5* | wild rice | nivara | - |
| *O. nivara-6* | wild rice | nivara | - |
| *O. nivara-7* | wild rice | nivara | Laos |
| *O. rufipogon-10* | wild rice | rufipogon | Laos |
| *O. rufipogon-12* | wild rice | rufipogon | - |
| *O. rufipogon-15* | wild rice | rufipogon | - |
| *O. rufipogon-16* | wild rice | rufipogon | China |
| *O. rufipogon-18* | wild rice | rufipogon | China |
| *O. rufipogon-19* | wild rice | rufipogon | China |
| *O. rufipogon-20* | wild rice | rufipogon | China |
| *O. rufipogon-21* | wild rice | rufipogon | China |
| Unknown* | landrace | indica | Taiwan |
| Unknown 3* | landrace | indica | Taiwan |
| Unknown 1* | landrace | japonica | Taiwan |

* Three unknown landrace accessions were collected from Taiwan.

^#^ landraces were collected from Taiwenese indigenous groups.

**Additional file 1: Table S2. Chromosomal position, locus name and PIC value of 75 SSR marker used for this study.**

| **Chr.** | **Position^a^** | **Locus^b^** | | **PIC^e^** | |  | **Chr.** | | **Position** | | | **Locus** | | **PIC** |  |
| --- | --- | --- | --- | --- | --- | --- | --- | --- | --- | --- | --- | --- | --- | --- | --- |
| 1 | 10.9 | STS322^C^ | (5) | 0.61 |  | | | 7 | | 11 | RM5055 | | (15) | 0.68 | |
| 1 | 49.6-50.8 | RM580 | (14) | 0.78 |  | | | 7 | | 31 | RM8010 | | (17) | 0.86 | |
| 1 | 135.8 | RM212 | (7) | 0.70 |  | | | 7 | | 41.7 | RM125 | | (6) | 0.44 | |
| 1 | 146.4 | RM472^d^ | (21) | 0.83 |  | | | 7 | | 61.9 | RM418 | | (17) | 0.87 | |
| 1 | 159.6 | RM3520 | (12) | 0.66 |  | | | 7 | | 99.6 | RM1364 | | (9) | 0.78 | |
| 2 | 4.7-6.9 | RM154^d^ | (17) | 0.79 |  | | | 7 | | 116.6 | CH0701 | | (12) | 0.72 | |
| 2 | 4.7-6.9 | RM6938 | (16) | 0.83 |  | | | 8 | | 1.9 | CH0880 | | (16) | 0.82 | |
| 2 | 36.3-36.8 | RM3390 | (5) | 0.18 |  | | | 8 | | 21.6-25.2 | SLS182 | | (4) | 0.46 | |
| 2 | 62.2 | RM1038 | (16) | 0.81 |  | | | 8 | | 60.1-60.4 | CH0868 | | (15) | 0.75 | |
| 2 | 101.5 | CH0219 | (12) | 0.76 |  | | | 8 | | 72.2 | RM515 | | (11) | 0.82 | |
| 2 | 157.9 | RM207 | (12) | 0.79 |  | | | 8 | | 99.1 | CH0877 | | (10) | 0.72 | |
| 3 | 1.1 | RM4108 | (28) | 0.90 |  | | | 8 | | 121.2 | RM4154 | | (34) | 0.95 | |
| 3 | 2.2 | RM3202^d^ | (15) | 0.85 |  | | | 9 | | 2.1 | CH0905 | | (6) | 0.68 | |
| 3 | 40.3 | RM218 | (8) | 0.80 |  | | | 9 | | 20.7 | RM219^d^ | | (16) | 0.87 | |
| 3 | 55.8 | RM282^d^ | (8) | 0.66 |  | | | 9 | | 26.7-30.6 | CH0919 | | (9) | 0.82 | |
| 3 | 59.5 | RM6929 | (15) | 0.85 |  | | | 9 | | 90.1 | RM6971 | | (12) | 0.74 | |
| 3 | 115.6 | RM2334 | (26) | 0.90 |  | | | 10 | | 23.1 | CH1007 | | (19) | 0.89 | |
| 3 | 120.1-120.4 | RM6736 | (9) | 0.70 |  | | | 10 | | 30.2 | RM5708^d^ | | (37) | 0.92 | |
| 3 | 120.4 | RM135^d^ | (5) | 0.48 |  | | | 10 | | 48.4 | CH1014 | | (14) | 0.80 | |
| 3 | 149.1-151.5 | RM143^d^ | (6) | 0.63 |  | | | 10 | | 55.6-57.5 | CH1016 | | (18) | 0.86 | |
| 4 | 18.3-19.6 | RM7472 | (17) | 0.76 |  | | | 11 | | 1.4 | RM1761 | | (33) | 0.90 | |
| 4 | 30.8 | CH0440 | (8) | 0.79 |  | | | 11 | | 19.8 | RM6544 | | (8) | 0.58 | |
| 4 | 49.7-52.6 | STS319^d^ | (4) | 0.39 |  | | | 11 | | 49.1 | RM536^d^ | | (9) | 0.81 | |
| 4 | 74.2 | CH0451 | (7) | 0.68 |  | | | 11 | | 57.3 | CH1106^d^ | | (9) | 0.43 | |
| 4 | 100.7 | CH0455 | (12) | 0.77 |  | | | 11 | | 64.8-68.4 | RM1355 | | (10) | 0.71 | |
| 4 | 114.3 | RM3648 | (10) | 0.54 |  | | | 11 | | 99.2-101.9 | CH1109 | | (5) | 0.69 | |
| 5 | 14.1 | RM1024 | (4) | 0.43 |  | | | 11 | | 115.1 | RM224 | | (11) | 0.82 | |
| 5 | 39.2 | STS204^d^ | (3) | 0.40 |  | | | 12 | | 0.0-5.5 | CH1210^d^ | | (5) | 0.55 | |
| 5 | 59.0-60.7 | CH0509 | (29) | 0.90 |  | | | 12 | | 10.8 | RM8215 | | (10) | 0.68 | |
| 5 | 73.9-75.0 | RM164 | (13) | 0.79 |  | | | 12 | | 27.1 | RM3472^d^ | | (19) | 0.80 | |
| 5 | 112.4-115.7 | RM3664 | (7) | 0.75 |  | | | 12 | | 38.1 | RM3455 | | (11) | 0.67 | |
| 5 | 122 | RM1054^d^ | (11) | 0.66 |  | | | 12 | | 41.2 | RM6905 | | (19) | 0.87 | |
| 6 | 13.8 | RM6734 | (12) | 0.69 |  | | | 12 | | 69.6 | RM519 | | (7) | 0.61 | |
| 6 | 33.5-34.3 | RM276 | (14) | 0.84 |  | | | 12 | | 91.3 | RM270^d^ | | (12) | 0.69 | |
| 6 | 83 | CH0607 | (4) | 0.54 |  | | | 12 | | 105.7-106.1 | CH0862^d^ | | (4) | 0.44 | |
| 6 | 85.4 | STS354^d^ | (10) | 0.65 |  | | | 12 | | 107.4 | RM17^d^ | | (13) | 0.51 | |
| 6 | 109.5-110.6 | CH0629 | (6) | 0.69 |  | | | 12 | | 109.2 | RM2197 | | (13) |  | |
| 6 | 124.4 | P17G10-24 | (30) | 0.94 |  | | |  | |  |  | |  |  | |

^a^ The position of each locus referred to a published linkage map from RGAP (Rice Genome Annotation Project).

^b^ The digit in brackets () indicates the marker allele number of 148 accessions.

^c^ RM - Rice Microsatellite (McCouch *et al*., 2002), STS - Sequence Tag Site (Ware *et al*., 2000), CH – InDel marker.

^d^ Marker amplified with multiplex PCR

^e^ PIC – polymorphism information content (Botstein, 1980)。
