# Supplementary material for: Genetic Diversity of Landraces and Improved Varieties of Rice (Oryza sativa L.) in Taiwan
Source: Rice (N Y). 2020 Dec 14;13:82. doi: 10.1186/s12284-020-00445-w (PMC7736384; doi:10.1186/s12284-020-00445-w)
Supplement: Supplementary file 2 — Additional file 2: Figure S1. The frequency distribution of allele number and polymorphic information content (PIC) with 75 molecular markers. (A) Allele number per locus ranges from 3 to 37 with an average of 12.7. (B) PIC ranges from 0.18 to 0.95 with an average of 0.72. Figure S2. (a) Structure simulation analysis to determine best K. (A) LnP(D), the log likelihood for each K, was calculated by 100,000 permutations and mean LnP(D) value was taken from 10 replications. ∆K, an ad hoc quantity, is transferred by mean LnP(D) value and ∆K of 148 accessions. (B) LnP(D) value and ΔK of 86 indica accessions. (C) LnP(D) value and ΔK of 86 japonica accessions. Figure S3. Population structure analysis of 148 accessions. Each individual is indicated by a vertical bar. (A) For K = 2, pop2–1 and pop2–2, indicated by red and green, are composed of japonica and indica rice, respectively. (B) For K = 5, pop5–1, pop5–2, pop5–3, pop5–4 and pop5–5, indicated by red, green, blue, yellow and magenta, are composed of indica cultivar, japonica landrace, japonica cultivar, indica landraces and wild rices, respectively. The numbers of accessions in each subpopulation are indicated in brackets (). Figure S4. Three-dimensional plot from principle coordinate analysis of 148 rice accessions. Japonica and indica are separated on opposite sides. Japonica and indica cultivars are marked with circles. [file 12284_2020_445_MOESM2_ESM.pptx]

## Slide 1
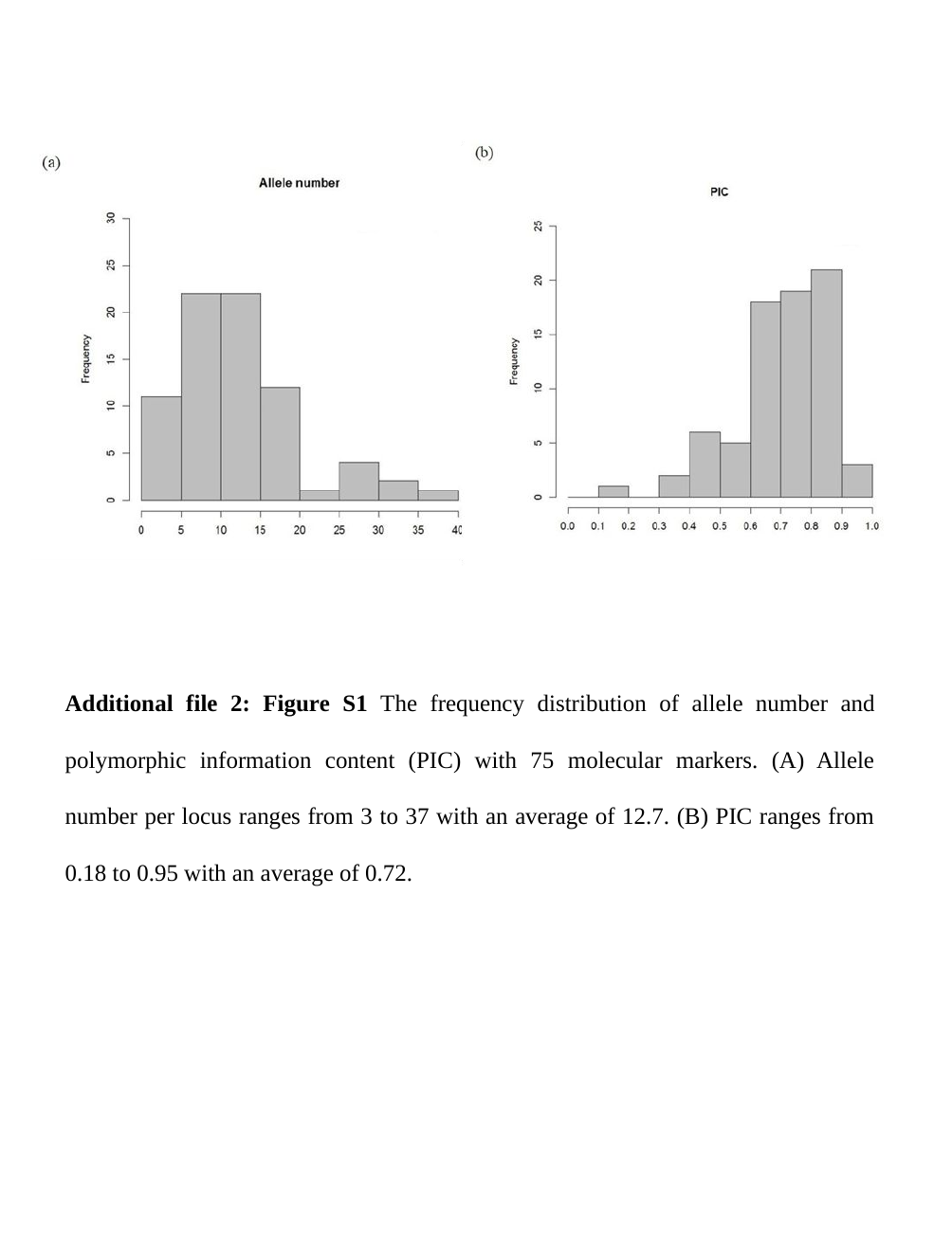

Additional file 2: Figure S1 The frequency distribution of allele number and polymorphic information content (PIC) with 75 molecular markers. (A) Allele number per locus ranges from 3 to 37 with an average of 12.7. (B) PIC ranges from 0.18 to 0.95 with an average of 0.72.

## Slide 2
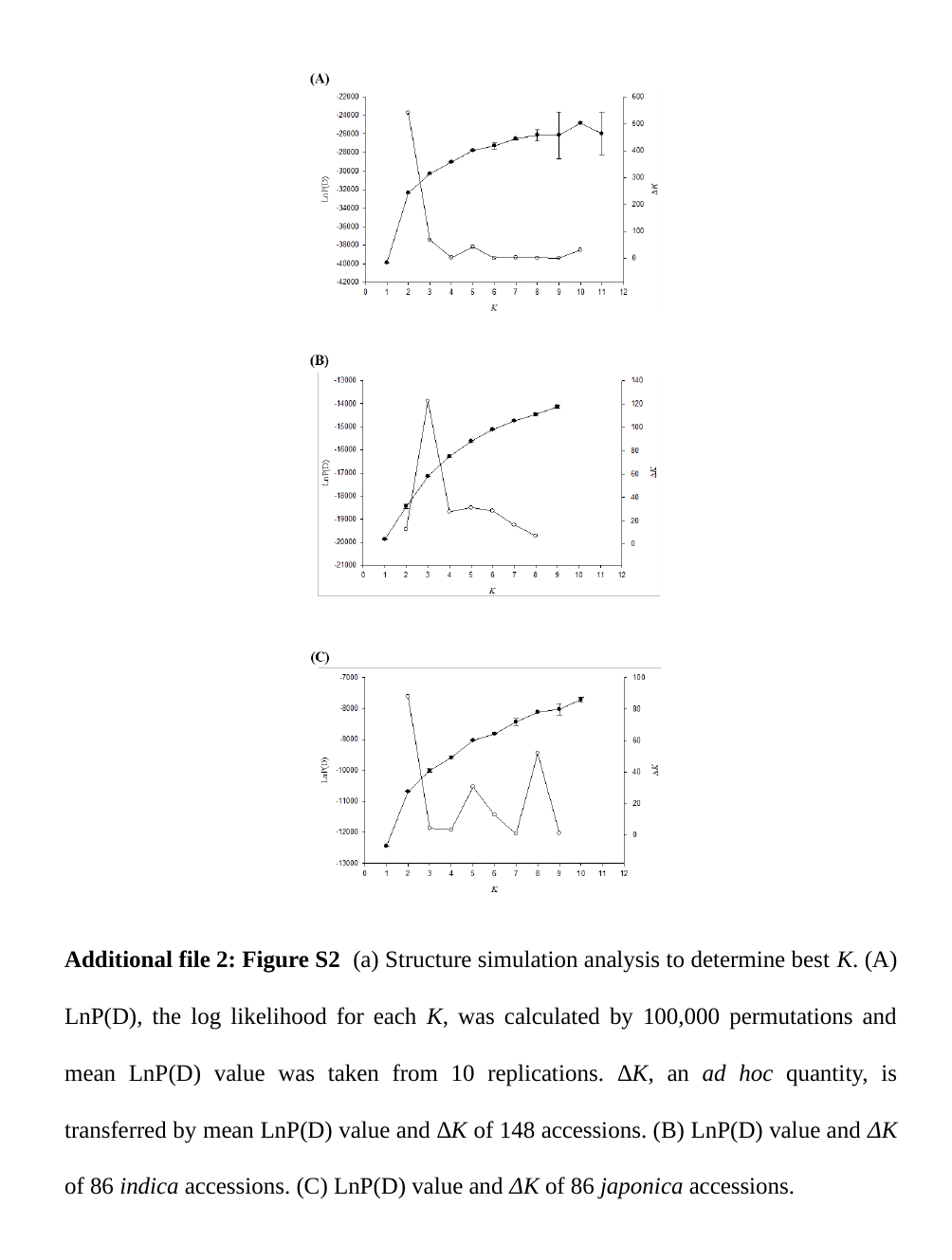

Additional file 2: Figure S2 (a) Structure simulation analysis to determine best K. (A) LnP(D), the log likelihood for each K, was calculated by 100,000 permutations and mean LnP(D) value was taken from 10 replications. ∆K, an ad hoc quantity, is transferred by mean LnP(D) value and ∆K of 148 accessions. (B) LnP(D) value and ΔK of 86 indica accessions. (C) LnP(D) value and ΔK of 86 japonica accessions.

## Slide 3
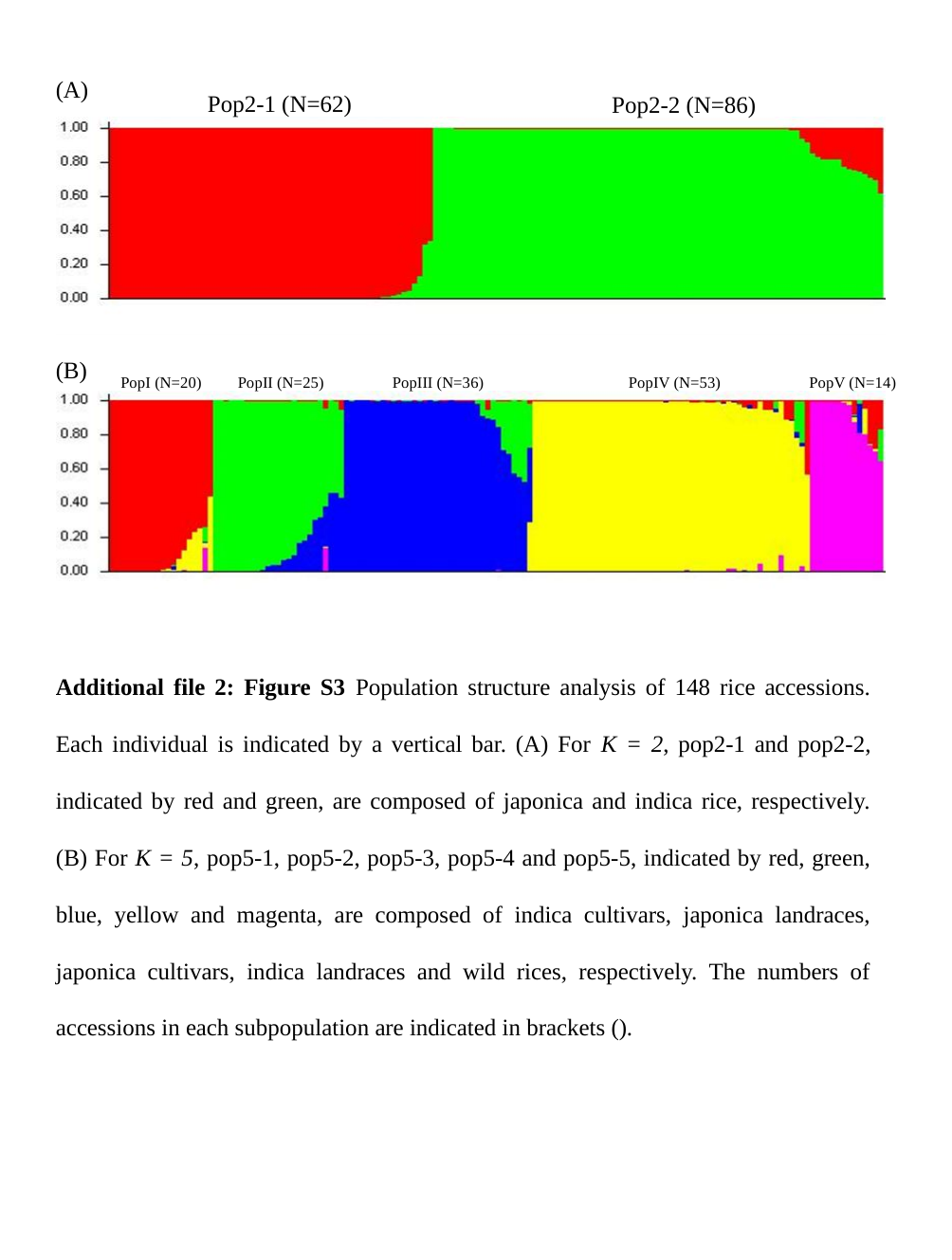

(A)
Pop2-1 (N=62)
Pop2-2 (N=86)
(B)
 PopI (N=20) PopII (N=25) PopIII (N=36) PopIV (N=53) PopV (N=14)
Additional file 2: Figure S3 Population structure analysis of 148 rice accessions. Each individual is indicated by a vertical bar. (A) For K = 2, pop2-1 and pop2-2, indicated by red and green, are composed of japonica and indica rice, respectively. (B) For K = 5, pop5-1, pop5-2, pop5-3, pop5-4 and pop5-5, indicated by red, green, blue, yellow and magenta, are composed of indica cultivars, japonica landraces, japonica cultivars, indica landraces and wild rices, respectively. The numbers of accessions in each subpopulation are indicated in brackets ().

## Slide 4
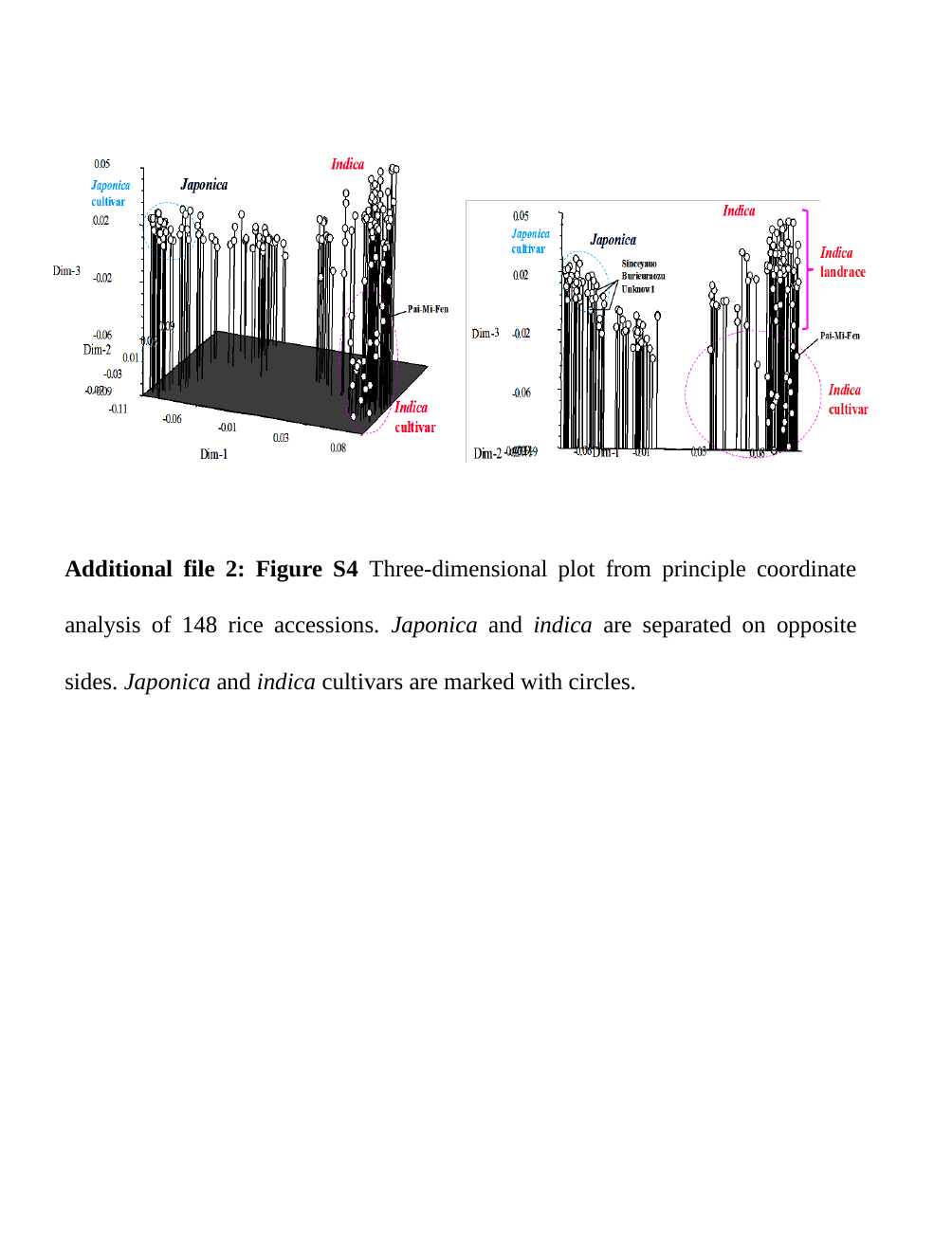

Additional file 2: Figure S4 Three-dimensional plot from principle coordinate analysis of 148 rice accessions. Japonica and indica are separated on opposite sides. Japonica and indica cultivars are marked with circles.
